# Causal associations of gut microbiota and pulmonary tuberculosis: a two-sample Mendelian randomization study

**DOI:** 10.3389/fmicb.2024.1400214

**Published:** 2024-06-14

**Authors:** Zhongkui Lu, Weiping Xu, Yidi Guo, Fang He, Guoying Zhang

**Affiliations:** Department of Clinical Laboratory, Nanjing Integrated Traditional Chinese and Western Medicine Hospital Affiliated with Nanjing University of Chinese Medicine, Nanjing, Jiangsu, China

**Keywords:** GM, PTB, MR, Dorea, *Parasutterella*, *Lachnoclostridium*

## Abstract

**Background:**

The prevalence of pulmonary tuberculosis (PTB) as an infectious disease continues to contribute significantly to global mortality. According to recent studies, the gut microbiota of PTB patients and healthy controls (HCs) show significant disparities. However, the causal relationship between them has yet to be elucidated.

**Methods:**

We conducted a study using Mendelian Randomization (MR) to explore the potential causal link between gut microbiota and pulmonary tuberculosis (PTB). The summary statistics of the gut microbiota were acquired from the MiBioGen consortium, while data on PTB were sourced from pheweb.jp. A range of statistical methodologies were employed to evaluate causality, encompassing inverse variance weighting (IVW), MR-Egger, weighted median (WM), weighted model, and simple model. We utilized instrumental variables (IVs) that have a direct causal relationship with PTB to annotate SNPs, aiming to discover the genes harboring these genetic variants and uncover potential associations between host genes and the microbiome in patients with PTB.

**Results:**

Among the 196 bacterial traits in the gut microbiome, we have identified a total of three microbiomes that exhibit a significant association with PTB. The occurrence of Dorea (*P* = 0.0458, FDR-adjusted *P* = 0.0458) and Parasutterella (*P* = 0.0056, FDR-adjusted *P* = 0.0168) was linked to an elevated risk of PTB, while the presence of Lachnoclostridium (*P* = 0.0347, FDR-adjusted *P* = 0.0520) demonstrated a protective effect against PTB. Our reverse Two-Sample Mendelian Randomization (TSMR) analysis did not yield any evidence supporting the hypothesis of reverse causality from PTB to alterations in the intestinal flora.

**Conclusion:**

We have established a connection between the gut microbiota and PTB through gene prediction analysis, supporting the use of gut microecological therapy in managing PTB and paving the way for further understanding of how gut microbiota contributes to PTB’s development.

## 1 Introduction

The etiological agent of tuberculosis is Mycobacterium tuberculosis, a pathogenic bacterium primarily transmitted through airborne routes (Sia and Rengarajan, 2019). According to the 2022 report by the World Health Organization on tuberculosis (TB), there were a total of 10.6 million diagnosed TB cases, resulting in approximately 1.13 million fatalities in the same year. However, the net reduction from 2015 to 2022 fell short of the WHO End TB Strategy milestone, which aimed for a 50% decrease by 2025, achieving only an 8.7% decline. Furthermore, it is projected that around 410,000 individuals (95% UI:370,000–450,000) were impacted by multidrug-resistant or rifampicin-resistant TB (MDR/RR-TB) in 2022 (Bagcchi, 2023). Moreover, drug-resistant Mtb and the increasing incidence of TB-HIV coinfection pose significant public health threats. The majority of TB cases in clinical practice are pulmonary tuberculosis (PTB), accounting for 80–90% (Dheda et al., 2017). PTB can lead to lung granuloma formation as well as tissue liquefaction and cavity development (Gupta et al., 2022).

The human gut microbiota consists of bacteria, archaea, viruses, and microbial eukaryotes, all of which play a pivotal role in maintaining human health (Cresci and Bawden, 2015). The gut microbiota can exert influence on the occurrence and progression of lung diseases through the modulation of circulating immune cells, inflammatory mediators, bacterial metabolites, and bacterial translocation, which is referred to as the gut-lung axis (Wang et al., 2023). Recent scientific studies indicate a possible link between the gut microbiome and the development of pulmonary tuberculosis (PTB) (Chhabra et al., 2021). Significant alterations have been observed in the gut microbiota composition of PTB patients compared to healthy individuals (Hu et al., 2019). In Burkina Faso, the growth of *M. tuberculosis* strains in vitro was found to be inhibited by two Firmicutes species, namely *E. casseliflavus* and *E. mundtii*, which were isolated from patients who tested negative for TB (Fellag et al., 2020). A study conducted in western China revealed a direct relationship between the gut microbiota and the number of peripheral CD4^+^ T cells in individuals with tuberculosis (Luo et al., 2017). Conversely, a study conducted in India found that individuals with tuberculosis had an increased presence of bacteria that produce butyrate and propionate (Hajian et al., 2019). Furthermore, during anti-TB chemotherapy treatment, both intestinal flora diversity and fecal metabolite abundance were restored among PTB patients (Meng et al., 2022). Although several studies have described associations between bacterial function changes and PTB (Li et al., 2019; Wang et al., 2022), the specific mechanisms underlying these relationships remain unclear.

The majority of pertinent studies are of an observational nature, rendering the establishment of causal conclusions a challenging endeavor. While in vitro experiments can elucidate specific mechanisms by which certain bacteria respond to PTB; identifying truly causative bacteria from thousands remains difficult using this approach alone. Mendelian randomization (MR) establishes causal relationships between exposure and outcomes by using single nucleotide polymorphisms (SNPs) as instrumental variables (IVs), simulating a randomized controlled trial. The present study employed a two-sample Mendelian randomization approach to investigate the potential causal association between gut microbiota and PTB, with gut microbiota as the exposure factor and PTB as the outcome variable, aiming to explore their genetic relationship.

## 2 Materials and methods

### 2.1 Study design

The Two-Sample Mendelian Randomization (TSMR) approach was employed to investigate the causal relationship between intestinal bacterial taxa and PTB. To ensure robust findings, three assumptions must be met when conducting TSMR analysis: (1) there is a significant association between genetic variations and factors of exposure; (2) genetic variations do not show any correlation with confounding variables; and (3) the influence of genetic variants on the outcome is solely through exposure factors, excluding any alternative pathways like horizontal pleiotropy. The genetic variants that satisfy these three assumptions can serve as instrumental variables in TSMR analysis (Emdin et al., 2017).

### 2.2 Data sources

The statistical data on the gut microbiota were obtained from a comprehensive genome-wide association study (GWAS) conducted by the MiBioGen consortium,^1^ which involved 18,340 participants across 24 cohorts (Kurilshikov et al., 2021). We eliminated 15 bacterial traits that lacked precise nomenclature, leading to a conclusive collection of 196 bacterial traits spanning across 9 Phyla, 16 Classes, 20 Orders, 32 Families, and 119 Genera. Data for PTB cases and controls were sourced from pheweb.jp,^2^ comprising a total of 170871 controls, 7800 cases and 12,454,677 SNPs.

### 2.3 Instrumental variables

The instrumental variables were identified in a genome-wide association study (GWAS) of intestinal flora using the following criteria: (1) to increase the number of SNPs as instrumental variables, we selected SNPs that showed correlation with the risk factors at a relatively lenient statistical significance threshold (*P* < 1 × 10^–5^) (Sanna et al., 2019; Li et al., 2022; Liu et al., 2022; Huang et al., 2023), (2) to eliminate SNPs in high linkage disequilibrium (R2 > 0.1) based on reference data from individuals of European ancestry obtained from the 500 Genomes project, and (3) to assess the strength of each SNP by calculating its F statistic and discard weak instrumental SNPs (*F* < 10).

### 2.4 Mendelian randomization analysis

Five popular Mendelian randomization (MR) methods were employed for analyzing valid instrumental variables (IVs): inverse variance-weighted (IVW) test, weighted median, MR-Egger regression, simple mode, and weighted mode. The IVW method was primarily used among various approaches due to its slightly higher statistical power in certain situations. It incorporated weights derived from the reciprocal of outcome variance for fitting purposes, regardless of whether an intercept term was included in the regression model. Complementary assessments were conducted using the remaining four methods, each relying on distinct assumptions regarding potential pleiotropy. Consistency between these complementary approaches and the IVW estimation results would strengthen the robustness of effect estimation.

The odds ratio (OR) and 95% confidence interval (CI) were computed as per standard procedures. A significance level of *P* < 0.05 was used to determine statistical significance. Additionally, we utilized the False Discovery Rate (FDR) correction method to assess the potential for multiple comparisons in our findings. A significant causal relationship was determined to be present when the adjusted P-value was less than 0.05. Conversely, if an unadjusted *p*-value was < 0.05 but the FDR-adjusted p-value was > 0.05, it was considered as suggestive evidence of a potential association (Zhang et al., 2024). The FDR method is detailed as follows: (1) Extract the p-values calculated using the same method. (2) List the p-values in ascending order. (3) Adjust the p-value according to the formula (adjusted *P*-value = *P*-value * (Total number of P-values / Ranking number of the current *P*-value)). The presence of heterogeneity among SNPs was evaluated using Cochran’s Q statistic (Cohen et al., 2015). The absence of heterogeneity is suggested when the *P*-value exceeds 0.05. To evaluate horizontal pleiotropy, we employed the MR-PRESSO test in conjunction with the intercept derived from MR-Egger regression (Bowden et al., 2015). The absence of horizontal pleiotropy was indicated by a *P*-value greater than 0.05. To ensure the robustness of the findings, a sensitivity analysis was conducted using the leave-one-out method (Wu et al., 2020). All tests were conducted using the R software version 4.3 and employed the TwoSampleMR package in R, employing a two-sided approach.

## 3 Results

### 3.1 The selection of instrumental variables

We screened the instrumental variables of 196 bacteria, excluding 15 unidentified genera. A total of 13,659 instrumental variables achieved locus-wide significance (*p* < 1 × 10^–5^). After accounting for linkage disequilibrium effects specific to flora, we retained 2708 instrumental variables. Subsequently, we excluded weakly associated exposure factors (*F* < 10) and potential confounding factors from outcomes analysis, resulting in a final inclusion of 1708 instrumental variables representing the diverse flora.

### 3.2 Causal effects from gut microbiota on PTB risk

**FIGURE 1:**
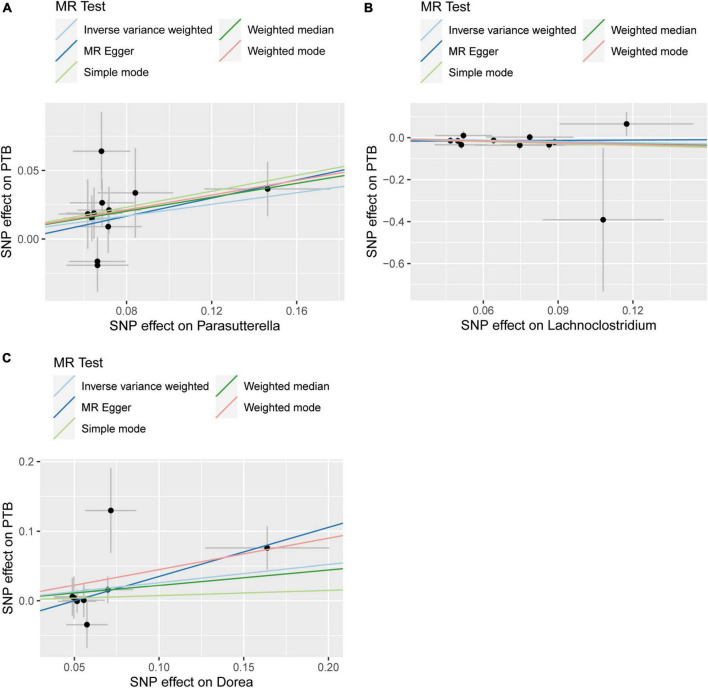
Summary of scatter plots of potential associations between gut microbiota and plumonary tuberculosis (PTB). **(A)** Potential positive associations between Parasutterella and PTB, **(B)** potential negative associations between Lachnoclostrdium and PTB, **(C)** potential positive associations between Dorea and PTB.

**FIGURE 2:**
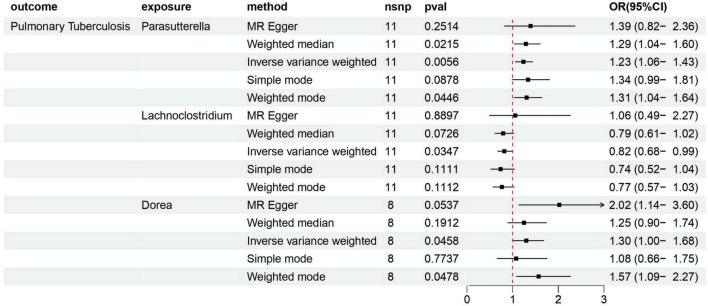
Forest plot of the causality between 3 bacterial taxa with the risks of PTB.

According to various Mendelian randomization (MR) analysis methods, we have obtained compelling evidence suggesting a potential causal relationship between the gut microbiota and the risk of PTB, with statistical significance set at 0.05 (Figure 1). The instrumental variable weighted (IVW) analysis revealed that Lachnoclostridium (OR = 0.82, 95%CI = 0.68 ∼ 0.99, P = 0.0347) exhibited a protective effect against PTB. Furthermore, Parasutterella (OR = 1.23, 95%CI = 1.06 ∼ 1.43, *P* = 0.0058) and Dorea (OR = 1.30, 95%CI = 1.00 ∼ 1.68, *P* = 0 .0458) were found to be inversely associated with PTB (Figure 2). Based on the FDR correction results, it has been determined that there is a significant causal relationship between Parasutterella (adjusted *P* = 0.0168) and Dorea (adjusted *P* = 0.0458) with PTB. Additionally, it has been found that Lachnoclostridium (adjusted *P* = 0.0520) may have a potential causal relationship with PTB (Table 1).

**TABLE 1:** False Discovery Rate (FDR) correction.

Outcome	Exposure	Method	*P*-value	FDR-adjusted *P*-value
PTB	*Parasutterella*	MR Egger	0.2514	0.3772
		Weighted median	0.0215	0.0645
		Inverse variance weighted	0.0056	0.0168
		Simple mode	0.0878	0.2635
		Weighted mode	0.0446	0.1337
	*Lachnoclostridium*	MR Egger	0.8897	0.8897
		Weighted median	0.0726	0.1089
		Inverse variance weighted	0.0347	0.0520
		Simple mode	0.1111	0.1667
		Weighted mode	0.1112	0.1112
	Dorea	MR Egger	0.0537	0.1612
		Weighted median	0.1912	0.1912
		Inverse variance weighted	0.0458	0.0458
		Simple mode	0.7737	0.7737
		Weighted mode	0.0478	0.0717

### 3.3 Sensitivity analysis

**FIGURE 3:**
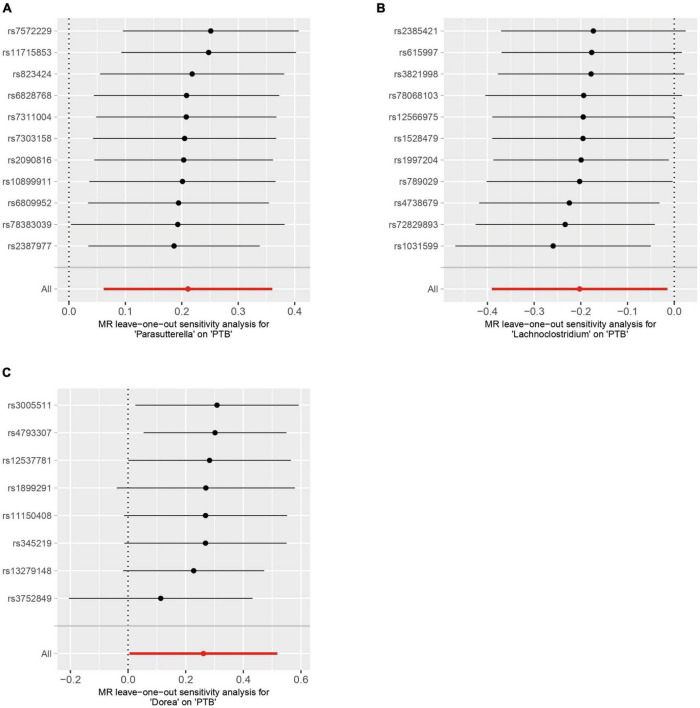
Sensitivity analysis by using the leave-one-out-method. **(A)** Parasutterella on PTB, **(B)** Lachnoclostridium on PTB **(C)** Dorea on PTB.

The Cochran’s Q test results indicated no evidence of heterogeneity among the independent variables (Table 2). The MR-Egger intercept test, excluding outlier variants, demonstrated the absence of horizontal pleiotropy (Table 3). Sensitivity analysis using the leave-one-out method confirmed the stability of our findings when systematically removing individual SNPs (Figure 3).

**TABLE 2:** Evaluation of heterogeneity using different methods.

Outcome	Exposure	Method	Cochran’s Q	*P*-value
PTB	*Parasutterella*	MR Egger	9.75	0.37
		Inverse variance weighted	9.98	0.44
	*Lachnoclostridium*	MR Egger	7.90	0.54
		Inverse variance weighted	8.37	0.59
	Dorea	MR Egger	5.11	0.53
		Inverse variance weighted	7.86	0.34

**TABLE 3:** Evaluation of horizontal pleiotropy.

Outcome	Exposure	Egger-intercept	SE	*P*-value
PTB	*Parasutterella*	−0.01	0.02	0.65
	*Lachnoclostridium*	−0.02	0.03	0.51
	Dorea	−0.04	0.02	0.15

### 3.4 The result of reverse MR analysis

Subsequently, we conducted reverse Mendelian randomization (MR) analyses to assess the potential inverse associations between three bacterial traits and PTB. However, the IVW approach did not find any significant associations between PTB and the mentioned bacterial traits (OR: 0.94; 95% CI: 0.87 ∼ 1.03; *p* = 0.216 for Dorea; OR: 0.98; 95% CI: 0.84 ∼ 1.13; *p* = 0.797 for Parasutterella and OR: 1.05; 95% CI: 0.96 ∼ 1.15; *p* = 0.285 for Lachnoclostridium) (Table 4).

**TABLE 4:** Reverse MR analysis by using the IVW method.

Bacterium	Method	OR (95%CI)	*P*-value
*Parasutterella*	Inverse variance weighted	0.98(0.84 ∼ 1.13)	0.797
*Lachnoclostridium*	Inverse variance weighted	1.05(0.96 ∼ 1.15)	0.285
Dorea	Inverse variance weighted	0.94(0.87 ∼ 1.03)	0.216

## 4 Discussion

This study systematically investigates the causal relationship between gut microbiota and PTB using Mendelian randomization. Our findings suggest a significant association between Parasutterella and Dorea with an increased risk of PTB, while indicating that Lachnoclostridium may potentially exert a protective effect against PTB. The robustness of these associations is further confirmed through sensitivity analysis. Importantly, no evidence supporting causal effects of other intestinal flora on PTB was identified in our study.

The human gut microbiome (GM) plays a pivotal role in maintaining human health, including the regulation of lung diseases. The gut microbiota and the lungs communicate through soluble microbial components and metabolites that are transported via circulation. Administering lipopolysaccharide (LPS) intrarectally to mice treated with antibiotics restored their capacity to mount efficient immune responses against influenza virus infection in the lungs (Ichinohe et al., 2011). Short-chain fatty acids (SCFAs), including butyrate, propionate, and acetate, are synthesized by the microbiota in the cecum and colon, exerting an influence on local intestinal immunity and immune cell development. Scientific evidence suggests that consumption of SCFAs can ameliorate pulmonary allergic reactions (Trompette et al., 2014; Cait et al., 2018) and confer protection against influenza virus infection (Steed et al., 2017). Additionally, immune cells possess the ability to migrate directly from the intestine to the respiratory tract through circulation, thereby shaping the immune microenvironment within the lungs. Consequently, the concept of a gut-lung axis was proposed.

The gut-lung axis may represent a potential mechanism underlying the occurrence and progression of pulmonary tuberculosis (PTB). Notably, studies have demonstrated significant differences in both diversity and taxonomic composition of the intestinal microbiota between PTB patients and healthy controls (HCs) (Ye et al., 2022). Furthermore, variations in gut microbiota profiles have been observed among different populations with PTB. A study conducted in western China revealed that Actinobacteria and Proteobacteria were significantly enriched in the feces of recurrent tuberculosis patients (RTB). Conversely, the phylum Bacteroidetes, which contains a variety of beneficial commensal organisms, was reduced (Luo et al., 2017). A study conducted in western China revealed that Actinobacteria and Proteobacteria were significantly enriched in the feces of recurrent tuberculosis patients (RTB). Conversely, the phylum Bacteroidetes, which contains a variety of beneficial commensal organisms, was reduced (Ye et al., 2022). At the same time, family *Veillonellacea* and *Bateroidaceae* and their genera *Veillonella* and *Bacteroides* significantly increased in the gut microbiota during anti-TB chemotherapy (Meng et al., 2022). However, it is important to note that these studies do not establish a causal relationship between gut microbiota and pulmonary tuberculosis. Although certain specific gut microbial species including E. casseliflavus and E. mundtii have shown inhibitory effects on Mycobacterium tuberculosis growth in vitro (Fellag et al., 2020), this does not necessarily imply a direct causal relationship in vivo.

Our study identified a causal association between three gut microbiota, namely Parasutterella, Dorea, and Lachnoclostridium, and PTB. Notably, Parasutterella has been found to have a causal relationship with various other conditions in several Mendelian randomization studies, including COVID-19 (Chen H. et al., 2023), intrahepatic cholangiocarcinoma (ICC) (Chen Z. et al., 2023), chronic kidney disease (CKD) (Ren et al., 2023), and prostatitis (Shen et al., 2024). Meanwhile, Dorea is associated with autism spectrum disorder (ASD) (Li Z. et al., 2023), inflammatory bowel disease (IBD) (Zhang et al., 2021), hypertensive disorders in pregnancy (HDP) (Wu et al., 2023a), polycystic ovary syndrome (PCOS) (Min et al., 2023), hemorrhagic stroke (Shen et al., 2023), age-related macular degeneration (AMD) (Li and Lu, 2023), nicotine dependence (Chen Y. et al., 2023), and insomnia (Li Z. et al., 2023). Similarly, Lachnochlostrium is linked to multiple diseases, including Alzheimer’s disease (AD) (Ning et al., 2022), nonalcoholic fatty liver disease (NAFLD) (Zhang et al., 2023), myasthenia gravis (Su et al., 2023), type 2 diabetes (Li and Li, 2023), gestational diabetes mellitus (Wu et al., 2023b), renal cell cancer (Yin et al., 2023), insomnia (Li Z. et al., 2023), peripheral artery disease (Shi et al., 2024), peripheral atherosclerosis (Jiang et al., 2023), Primary open-angle glaucoma (POAG) (Zhou et al., 2024), and intervertebral disc degeneration (Zheng et al., 2023). These studies demonstrate a causal association between gut microbiota and diseases. Mechanistically, colonization of the gastrointestinal tract by Parasutterella, without altering bacterial composition, leads to modifications in microbial-derived metabolites such as aromatic amino acids, bilirubin, purine derivatives, and bile acid derivatives (Ju et al., 2019). Lachnoclostridium utilizes cutC/D to convert choline into TMA, thereby promoting the development of atherosclerosis (Cai et al., 2022). Dorea serves as an indicator of immune activation and is positively correlated with T-cell production of IFN-γ (Riazati et al., 2022). Collectively, these findings underscore the pivotal roles played by these gut microbiotas in disease progression.

In conclusion, our study establishes a causal relationship between three specific gut microbiota and pulmonary tuberculosis, thereby offering novel insights into the investigation of the association between gut microbiota and pulmonary tuberculosis. They may modulate pulmonary tuberculosis by facilitating the circulation of immune cells, cytokines, bacterial derivatives, and other factors. However, the underlying mechanism remains unclear. The elucidation of the pathway linking intestinal flora and pulmonary tuberculosis necessitates further in vivo and in vitro experiments. Additionally, it should be noted that these findings are derived from the Japanese population and may not necessarily be representative of other ethnicities such as those in Europe and Africa. Lastly, due to insufficient detailed clinical information, subgroup analyses based on age and gender were not feasible.

## 5 Conclusion

In contrast to other studies, our investigation specifically identified three gut microbiota species that are causally associated with tuberculosis using a two-sample Mendelian randomization approach. These include one protective bacterium and two bacteria associated with an increased risk of PTB. These findings support the hypothesis that the gut microbiota plays a role in the development of PTB. Furthermore, the findings offer valuable perspectives and guidance for future mechanistic investigations, including the utilization of animal models or human trials based on biomarkers, which can facilitate the advancement and clinical application of potential microbiota-centered strategies against tuberculosis.

## Data availability statement

The datasets presented in this study can be found in online repositories. The names of the repository/repositories and accession number(s) can be found in the article/supplementary material.

## Ethics statement

The studies involving humans were approved by the Ethics Committee of Nanjing Integrated Chinese and Western Medicine Hospital. The studies were conducted in accordance with the local legislation and institutional requirements. Written informed consent for participation was not required from the participants or the participants’ legal guardians/next of kin because The data in this study came from public database and did not involve patient samples. Therefore, written informed consent was not required for this study.

## Author contributions

ZL: Writing−original draft. WX: Writing−original draft, Methodology. YG: Writing−original draft, Formal analysis. FH: Writing−original draft, Data curation. GZ: Writing−review and editing, Writing−original draft.

## Footnotes

1
https://mibiogen.gcc.rug.nl

2
https://pheweb.jp/pheno/PTB
